# TLR signaling in mast cells: common and unique features

**DOI:** 10.3389/fimmu.2012.00185

**Published:** 2012-07-04

**Authors:** Hilary Sandig, Silvia Bulfone-Paus

**Affiliations:** ^1^ Faculty of Human and Medical Sciences, University of Manchester,Manchester, UK; ^2^ Department of Immunology and Cell Biology, Research Center Borstel,Borstel, Germany

**Keywords:** cytokine, innate, mast cells, review, signaling, TLRs

## Abstract

In addition to the well known role of mast cells in immunity to multi-cellular parasites and in the pathogenesis of allergy and asthma, the importance of mast cells in the immune defense against bacteria and viruses is increasingly being recognized. Their location in the skin, gut, and airways puts mast cells in an ideal location to encounter and respond to pathogens, and in order to perform this function, these cells express a variety of pattern recognition receptors, including Toll-like receptors (TLRs). Mast cells respond to TLR ligands by secreting cytokines, chemokines, and lipid mediators, and some studies have found that TLR ligands can also cause degranulation, although this finding is contentious. In addition, stimulation via TLR ligands can synergize with signaling via the FcεRI, potentially enhancing the response of the cells to antigen *in vivo*. A great deal is now known about TLR signaling pathways. Some features of these pathways are cell type-specific, however, and work is under way to fully elucidate the TLR signaling cascades in the mast cell. Already, some interesting differences have been identified. This review aims to address what is known about the responses of mast cells to TLR ligands and the signaling pathways involved. Given the location of mast cells at sites exposed to the environment, the response of these cells to TLR ligands must be carefully regulated. The known mechanisms behind this regulation are also reviewed here.

## INTRODUCTION

Host cells utilize a variety of germline encoded receptors termed pathogen recognition receptors (PRRs), including the Toll-like receptors (TLR) and nucleotide-binding oligomerization domain (NOD) proteins, to recognize pathogens. These receptors allow the innate immune system to identify invading bacteria by their expression of pathogen-associated molecular patterns (PAMPs; Akira et al., 2006). Signaling via these receptors guides the immune system to mount the correct response to an invading pathogen, or to a harmless commensal, by a process which is not well understood (Blander and Sander, 2012).

Mast cells have traditionally been known for their roles in allergy and immunity to multi-cellular parasites (Metcalfe et al., 1997) but increasingly the crucial roles that they play in immune defense against bacteria and viruses are being recognized (Marshall, 2004; Abraham and St John, 2010). Mast cells are able to recognize pathogens via their expression of PRRs and by binding to antibodies with the FcRs (Abraham and St John, 2010). This review will focus on TLR expression, function and signaling, since the TLRs are the best studied PRR on mast cells.

The TLRs are a family of receptors which recognize a wide variety of PAMPs, as summarized in **Table 1**. Furthermore, it is increasingly being recognized that certain endogenous molecules which are expressed during tissue damage or disease are also TLRs agonists (Kawai and Akira, 2010). There are 10 human TLRs, TLR1–TLR10, while 13 are found in the murine genome, TLR1–TLR9 and TLR11–TLR13 (Lee et al., 2012). The receptors largely function as homodimers, with the exception of TLR2 which forms heterodimers with both TLR1 and TLR6 (Akira et al., 2006; Lee et al., 2012). The TLR2 homodimers and heterodimers are located on the cell surface, as are TLR4 and TLR5, while TLR3 and TLR7–TLR9 are endosomally located, allowing them to recognize intracellular nucleic acids (Lee et al., 2012). Among other ligands, TLR4 recognizes LPS, TLR5 binds flagellin, and TLR2 heterodimers recognize various lipopeptides (Akira et al., 2006; Lee et al., 2012; **Table 1**).

**Table 1:** The mainTLR ligands (adapted from Akira et al., 2006; Lee et al., 2012).

TLR	Physiological ligands	Synthetic ligands
TLR1-2	Triacylated lipopeptides (bacteria and mycobacteria)	Parri_3_CSK_4_
TLR2	Peptidoglycan (gram positive bacteria), phospholipomannan (*Candida albicans*), tGPI-mucins (*Trypanosoma*), haemagglutinin (measles virus), porins (*Neisseria*), lipoarabinomannan (mycobacteria), glucuronoxylomannan (*Cryptococcus neoformans*), HMGB1 (host)	
TLR2-6	Diacylated lipopeptides (*Mycoplasma*), LTA (Group B *Streptococcus*), zymosan (*Saccharomyces cerevisiae*)	FSL1, MALP-2, Parri_2_CSK_4_
TLR3	dsRNA (viruses)	Polyl:C
TLR4	LPS (Gram-negative bacteria), VSV glycoprotein G, RSV fusion protein, MMTV envelope protein, mannan (*Candida albicans*), glucuronoxylomannan (*Cryptococcus neoformans*), glycosylinositolphospholipids (*Trypanosoma*), HSP60, HSP70, fibrinogen, HMGB1 (all host proteins), nickel	
TLR5	Flagellin (Flagellated bacteria)	
TLR7	ssRNA (RNA viruses)	Imidazoquinoline compounds: imiquimod, resiquimod, loxoribine, R848
TLR8	ssRNA(RNA viruses)	Resiquimod
TLR9	CpG-DNA (bacteria and mycobacteria), DNA (viruses), haemozoin (*Plasmodium*)	CpG-A, CpG-B and CpG-C ODNs

Much work has been carried out to determine the signaling pathways triggered by the TLR receptors and the consequences of their ligation (Lu et al., 2008; Akira, 2009; Kawai and Akira, 2010). This review aims to address the ability of mast cells to respond to TLR ligands and to examine what is known about TLR signaling and its regulation in mast cells. In addition, cross-talk between the TLR signaling pathways and that of FcεRI has been identified (Avila and Gonzalez-Espinosa, 2011), and the mechanisms and consequences of this will be discussed.

## TLR EXPRESSION ON MAST CELLS

Several studies have been undertaken on murine and human mast cells isolated *ex vivo*, or differentiated from stem cells, as well as on mast cell lines to establish which TLRs are expressed. The findings of these studies are summarized in **Table 2**. The TLRs appear to be widely expressed by murine mast cells, with expression of TLR1–4 and 6–9 identified at least at the mRNA level (McCurdy et al., 2001; Supajatura et al., 2001; Masuda et al., 2002; Ikeda and Funaba, 2003; Matsushima et al., 2004; Li et al., 2009; Mrabet-Dahbi et al., 2009). Expression of TLR5 has not been demonstrated on murine mast cells (McCurdy et al., 2001; Supajatura et al., 2001; Ikeda and Funaba, 2003; Matsushima et al., 2004).

**Table 2:** TLR expression by mast cells.

	Murine	Human	Cell line
TLR1	+ BMMC mRNA^1^	- lung mRNA^2^	+ HMC-1 mRNA and protein^3^
	+ FSDMC mRNA^1^	- skin mRNA^2^	- LAD2 mRNA and protein^3,^ ^5^
		+ PBDMC mRNA and protein^3^	+ LAD2 protein^6^
		+ CBDMC mRNA^4^	
TLR2	+ BMMC mRNA^1-^ ^6-^^9^	+ lung mRNA^2^	- HMC-1 mRNA and protein^3,^ ^11^
	+ FSDMC mRNA^1^	+ lung protein^5^	+ LAD2 mRNA and protein^3,^ ^5,^ ^11^
	+ BMMC protein^9^	+ skin mRNA^2^	+ MC-9 mRNA^7^
	+ PCDMC protein^9^	+ PBDMC mRNA and protein^3^	
		+ CBDMC mRNA^4^	
		+ CBDMC mRNA and protein^10^	
		+ in polyps by IHC^4^	
TLR3	+ BMMC mRNA^1^	+ lung mRNA^2^	+ LAD1 protein^2^
	+ FSDMC mRNA^1^	+ skin mRNA^2^	+ HMC-1 mRNA and protein^3^
		+ PBDMC mRNA and protein^3^	+ LAD2 mRNA and protein^3^
		+ Bone marrow^3^	
TLR4	+ BMMC mRNA^1^’^6-^ ^9^’^12^	+ lung mRNA^2^	+ MC-9 mRNA^7-^ ^12^
	+ BMMC protein^1-^ ^9-^ ^13^	+ skin mRNA^2^	- HMC-1 mRNA and protein^3^
	+ FSDMC mRNA and protein^1^	+ PBDMC mRNA and protein^3,^ ^14^	+ HMC-1 mRNA and protein^11^
	+ peritoneal protein^1^	+ CBDMC mRNA and protein^10^	+LAD2 mRNA and protein^3,^ ^5,^ ^11^
	+ PCDMC protein^9^	- CBDMC mRNA^4^	
TLR5	- BMMC mRNA^1-^ ^6-^ ^8^	+ lung mRNA^2^	+ HMC-1 mRNA and protein^3^
	- FSDMC mRNA^1^	+ skin mRNA^2^	+ LAD2 mRNA and protein^3,^ ^5^
		+ PBDMC mRNA and protein^3^	- MC-9 mRNA^7^
TLR6	+ BMMC mRNA^1-^ ^6-^ ^9^	- lung mRNA^2^	+ HMC-1 mRNA and protein^3^
	+ FSDMC mRNA^1^	- skin mRNA^2^	+ LAD2 mRNA and protein^3^
		+ PBDMC mRNA and protein^3^	+ MC-9 mRNA^7^
		+ CBDMC mRNA^4^	
TLR7	+ BMMC mRNA^1^	+ lung mRNA^2^	+ HMC-1 mRNA and protein^3^
	+ FSDMC mRNA^1^	+ skin mRNA^2^	+ LAD2 mRNA and protein^3^
		+ PBDMC mRNA and protein^3^	
TLR8	+ BMMC mRNA^8^	- lung mRNA^2^	+ HMC-1 mRNA and protein^3^
		- skin mRNA^2^- PBDMC mRNA and	protein^3^ - LAD2 mRNA and protein^3^
TLR9	- BMMC mRNA^1^	+ lung mRNA^2^	+ HMC-1 mRNA and protein^3^
	+ FSDMC mRNA^1^	- skin mRNA^2^	+ LAD2 mRNA and protein^3,^ ^5^
	+ peritoneal protein^1^	+ PBDMC mRNA and protein^3^	
TLR10	No murine homolog	+ lung mRNA^2^	None tested
		+ skin mRNA^2^	

*TLR expression on mast cells is summarized. The references are indicated by superscript numbers:*

1 Matsushima et al., 2004;

2 Kulka and Metcalfe, 2006;

3 Kulka et al., 2004;

4 McCurdy et al., 2003;

5 Yoshioka et al., 2007;

6 Ikeda and Funaba, 2003;

7 McCurdy et al., 2001;

8 Supajatura et al., 2001;

9 Mrabet-Dahbi et al., 2009;

10 Varadaradjalou et al., 2003;

11 Kubo et al., 2007;

12 Masuda et al., 2002.

Expression of TLR1–10 with the exception of TLR8 has been identified on human mast cells, although some studies were unable to identify TLR1, 4, 6, or 9 (McCurdy et al., 2003; Okumura et al., 2003; Varadaradjalou et al., 2003; Kulka et al., 2004; Kulka and Metcalfe, 2006; Yoshioka et al., 2007). TLR expression on the mast cell lines LAD2, HMC-1, and MC-9 has been assessed with varied results in different studies (McCurdy et al., 2001; Masuda et al., 2002; Kulka et al., 2004; Kulka and Metcalfe, 2006; Kubo et al., 2007; Yoshioka et al., 2007). It should be noted that several receptors have only been detected at the mRNA level and that further work will be required to demonstrate protein expression.

The expression of TLR2 by mast cells has been studied in more detail and it has been suggested that bone marrow-derived mast cell (BMMC) do not express the whole TLR2 protein but rather a truncated protein lacking the intracellular signaling domain (Mrabet-Dahbi et al., 2009). Despite this, a range of studies have determined that mast cells are able to respond to TLR2 ligands, as discussed below, and this may be due to the fact that the truncated TLR2 is still able to form heterodimers (Mrabet-Dahbi et al., 2009).

## MAST CELL RESPONSES TO TLR STIMULATION

### MAST CELL RESPONSES TO CELL SURFACE TLRs

**FIGURE 1:**
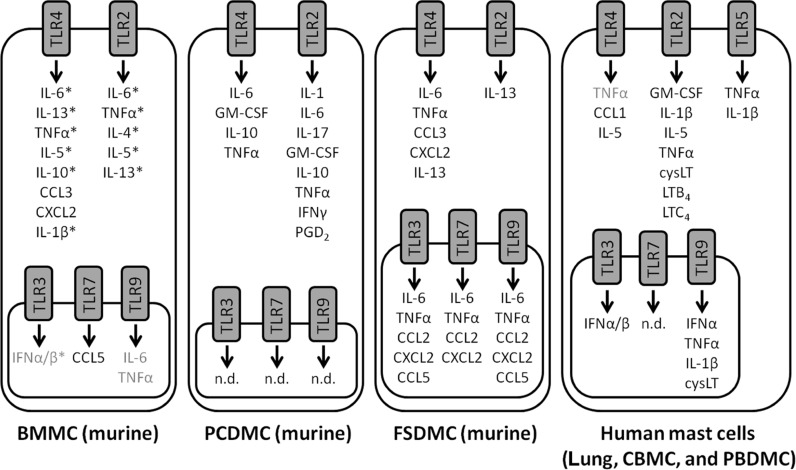
** Mast cell secretory responses to TLR ligation**. A diagram showing the molecules secreted by mast cells upon TLR ligation. The cytokines, chemokines, and lipid mediators released upon TLR ligand stimulation are summarized for murine BMMC, PCDMC, FSDMC, and human mast cells. Where there is discrepancy in the literature, molecules are shown in grey. *indicates instances where it has been demonstrated with the use of TLR-deficient cells or blocking antibodies that the ligand is acting via the indicated receptor.

Acting via TLR4, LPS caused IL-6, IL-13, and TNFα secretion from murine BMMC (McCurdy et al., 2001; Supajatura et al., 2001) and a later study found secretion of IL-5 and IL-10 upon LPS stimulation via TLR4 (Masuda et al., 2002). In addition to these cytokines, LPS stimulation of murine BMMC and fetal skin-derived mast cells (FSDMC) also caused the secretion of the chemokines CCL3/MIP-1α and CXCL2/MIP-2 (Matsushima et al., 2004; **Figure 1**).

Differences between the cytokines produced upon TLR4 and TLR2 stimulation have been observed: LPS caused murine BMMC to secrete TNFα, IL-6, IL-13, and IL-1β via TLR4; while peptidoglycan (PGN) causes the secretion of TNFα and IL-6, in addition to the Th2 cytokines, IL-4, IL-5, and IL-13 via TLR2 (Supajatura et al., 2002; **Figure 1**). In rat peritoneal mast cells, both PGN and LPS resulted in cysteinyl leukotriene production, but the response to PGN was greater (Wierzbicki and Brzezinska-Blaszczyk, 2009). Taken together, these findings suggest that mast cells release a wider variety of mediators in response to PGN than LPS. This appears not to be the case in macrophages, where stimulation with LPS or PGN has been shown to lead to an up-regulation of similar mRNAs (Wang et al., 2000).

Murine peritoneal cell-derived mast cells (PCDMC) responded more potently to TLR agonists than BMMC and it is suggested that the PCDMC are more mature than BMMC, and this increased maturity underlines their increased ability to respond to TLR stimulation (Mrabet-Dahbi et al., 2009). LTA and MALP-2 treatment of PCDMC resulted in IL-1, IL-6, IL-17, GM-CSF, IL-10, TNFα, and IFNγ production, while LPS caused only IL-6, GM-CSF, IL-10, and TNFα secretion from PCDMC (Mrabet-Dahbi et al., 2009). Of these three agonists, only LTA-induced PGD_2_ production in PCDMC (Mrabet-Dahbi et al., 2009; **Figure 1**).

In human cord blood-derived mast cells (CBMC), stimulation with zymosan or PGN caused GM-CSF, IL-1β, LTB_4_, and LTC_4_ production (Olynych et al., 2006). Another study also identified differences between the mediators released upon different TLR ligand stimulation: PGN, zymosan, and Pam_3_Cys caused GM-CSF and IL-1β secretion whereas LPS did not; and PGN and zymosan treatment led to the production of LTC_4_ unlike Pam_3_Cys treatment (McCurdy et al., 2003). Human mast cells cultured from CD34^+^ progenitors isolated from blood (PBDMC) stimulated with LPS produced significant amounts of TNFα, whereas PGN induced IL-1β, GM-CSF, IL-5, and cysteinyl leukotriene in addition to TNFα (Kulka et al., 2004; **Figure 1**). Therefore, as has been shown in murine mast cells, stimulation via TLR2 results in a greater range of mediator production than stimulation via TLR4.

Pre-treatment of mast cells with cytokines has been shown to enhance the response of the cells to TLR ligands (Okumura et al., 2003; Varadaradjalou et al., 2003). In one study, LPS only induced TNFα production after the CBMC had been incubated with IL-4, whereas even untreated cells were able to respond to PGN (Varadaradjalou et al., 2003). It is not clear why the cells in this study were unable to respond to the TLR4 agonist without TNFα pre-treatment (Varadaradjalou et al., 2003), unlike human PBDMC (Kulka et al., 2004). Human lung mast cells and PBDMC responded to LPS by secreting TNFα but this response, and TLR4 expression, was increased by pre-treatment with IFNγ (Okumura et al., 2003). This group also noticed CCL1 and IL-5 production in LPS-treated lung mast cells but not PBDMC, and gene array analysis showed that LPS caused the up-regulation of a variety of genes including a protease, several cytokines, chemokines, receptors, and STAT5a (Okumura et al., 2003).

Peptidoglycan has been demonstrated to induce migration of peritoneal rat mast cells after a short treatment with TNFα (Brzezinska-Blaszczyk and Rdzany, 2007), and in a later publication, LPS and PGN both caused migration of the cells after treatment with IL-6 or CCL5/RANTES, respectively (Wierzbicki and Brzezinska-Blaszczyk, 2009). The mechanism behind these effects is as yet unknown, but it has been suggested that IL-6 and CCL5/RANTES may modulate TLR expression on the mast cells (Wierzbicki and Brzezinska-Blaszczyk, 2009). The ability of TLR agonists to cause mast cell migration *in vivo* would allow PAMPs or endogenous TLR ligands produced upon tissue damage to recruit mast cells to sites of infection or inflammation.

TLR5 expression has been more readily detected on human than murine mast cells (see **Table 1**) and human PBDMC respond to flagellin (a TLR5 ligand) by secreting IL-1β and TNFα, demonstrating that the receptor is functional on these cells (Kulka et al., 2004; **Figure 1**). To our knowledge, flagellin has not been shown to cause cytokine secretion from murine mast cells, in agreement with the lack of detectable expression of TLR5 on the cells (McCurdy et al., 2001; Supajatura et al., 2001; Ikeda and Funaba, 2003; Matsushima et al., 2004).

### MAST CELL RESPONSES TO INTRACELLULAR TLRs

Double-stranded RNA molecules, such as polyI:C, are used as a synthetic mimic of viral RNA (see **Table 1**) and cause IFNα and β secretion from human PBDMC and murine BMMC (Kulka et al., 2004). This response was partially blocked with anti-TLR3 antibodies and in TLR3^-/-^ BMMC, suggesting that the receptor is involved in the detection of the RNA (Kulka et al., 2004). A different group found that murine FSDMC responded far more robustly to polyI:C than BMMC, secreting IL-6, TNFα, CCL2/MIP-1α, CXCL2/MIP-2, and CCL5/RANTES which was in agreement with the greater TLR3 expression by FSDMC than BMMC (Matsushima et al., 2004). In a recent study using BMMC, no IL-6, TNFα or IFNα/β production was observed upon polyI:C treatment (Keck et al., 2011; **Figure 1**). The ability of BMMC to respond to TLR3 stimulation is, therefore, somewhat controversial, although mast cells from other sources clearly do respond (Kulka et al., 2004; Matsushima et al., 2004). These finding may reflect the more immature phenotype of BMMC.

Murine BMMC have been found to respond to bacterial but not mammalian DNA, and to synthetic oligonucleotides containing an unmethylated cytosine followed by a guanosine (CpG motif), by secreting IL-6 and TNFα (Zhu and Marshall, 2001). MC/9 cells were also found to respond to CpG-containing oligonucleotides by secreting IL-6 and TNFα, and the response of BMMC was greater when greater numbers of CpG sequences were included in the oligonucleotides (Zhu and Marshall, 2001). These treatments were not found to induce mast cell degranulation or the secretion of GM-CSF, IL-4, IL-12, or IFNγ (Zhu and Marshall, 2001). A later study comparing BMMC and FSDMC found that TLR9 was expressed by FSDMC but not BMMC, and demonstrated TNFα, IL-6, CCL2/MIP-1α, CXCL2/MIP-2, and CCL5/RANTES secretion by FSDMC but not BMMC treated with CpG-containing oligonucleotides (Matsushima et al., 2004; **Figure 1**).

The TLR7 agonist, R848, caused secretion of IL-6 and TNFα and also the chemokines CCL2/MIP-1α and CXCL2/MIP-2 from FSDMC but not BMMC, and TLR7 expression was far higher in FSDMC (Matsushima et al., 2004). In spite of this, R848 stimulation of BMMC did lead to some CCL5/RANTES production (Matsushima et al., 2004; **Figure 1**), therefore murine mast cells appear to respond to TLR7 agonists, in agreement with their expression of the receptor (see **Table 2**).

A study using human PBDMC found that CpG-containing oligonucleotides stimulated cells to produce IFNα, IL-1β, TNFα, and cysteinyl leukotriene (Kulka et al., 2004). CpG-containing oligonucleotides activate TLR9 therefore these data suggest that in addition to expressing TLR9 (see **Table 2**), both human and murine mast cells are able to respond to TLR9 ligands by secreting cytokines and lipid mediators. The sensitivity of mast cells to TLR7 and TLR9 agonists would presumably assist in the immune defense against bacteria, viruses, and *Plasmodium* (see **Table 1**).

Fetal skin-derived mast cells express higher levels of TLR3, TLR7, and TLR9 and respond more potently to agonists of these receptors than BMMC (Matsushima et al., 2004), in a similar manner to the greater response of FSDMC than BMMC to TLR2 and 4 agonists (Mrabet-Dahbi et al., 2009). These results are likely a reflection of the immaturity of BMMC and suggest that responses to some TLRs are better studied in mast cell models other than BMMC (Matsushima et al., 2004; Mrabet-Dahbi et al., 2009).

Work performed in other immune cells has demonstrated that TLR3 and 9 signaling requires endosomal acidification and maturation, presumably because these receptors are intracellularly located (Ahmad-Nejad et al., 2002; Matsumoto et al., 2003; Akira, 2009). Similarly in FSDMC, the cytokine secretion induced by polyI:C (TLR3 ligand), R848 (TLR7 ligand), and CpG (TLR9 ligand) was inhibited by an inhibitor of endosomal maturation (Matsushima et al., 2004). In contrast, the mast cell response to LPS was unaffected by the treatment (Matsushima et al., 2004), in agreement with similar studies in a macrophage cell line (Ahmad-Nejad et al., 2002), presumably because TLR4 binds LPS at the cell surface.

### THE EFFECT OF TLR LIGATION ON MAST CELL DEGRANULATION AND PHENOTYPE

In addition to these findings that stimulation of mast cells with TLR agonists leads to cytokine and chemokine production and mast cell migration, some data suggest that mast cell degranulation can be induced by TLR2 ligands. Stimulation of BMMC with PGN resulted in mast cell degranulation whereas stimulation via TLR4 did not (Supajatura et al., 2002). Similar results were obtained in human mast cells (Varadaradjalou et al., 2003). Stimulation of CBMC with PGN led to histamine release in addition to cytokine release, whereas LPS stimulation caused only cytokine secretion (Varadaradjalou et al., 2003). In another study using human CBMC, the degranulation induced by PGN was not found to be statistically significant, while zymosan and Pam_3_Cys induced significant degranulation (McCurdy et al., 2003). *In vivo*, i.d. injection of PGN but not LPS caused a mast cell-dependent increase in vascular permeability, indicating that the TLR2 ligand was inducing mast cell degranulation (Supajatura et al., 2002).

Other groups have been unable to demonstrate degranulation after stimulation of mast cells with TLR ligands, however. Neither LPS nor PGN induced degranulation of BMMC (Ikeda and Funaba, 2003) or rat peritoneal mast cells (Wierzbicki and Brzezinska-Blaszczyk, 2009), and studying BMMC and FSDMC, Matsushima et al. (2004) did not detect degranulation in response to LPS, PGN, polyI:C, R848, or CpG, suggesting that signaling via TLR2–4, 7, and 9 does not cause mast cell degranulation. In agreement with these findings, TLR1/TLR2, TLR2/TLR6, and TLR4 agonists were not observed to cause degranulation of MC/9 cells or BMMC in another study (Qiao et al., 2006). Degranulation was not observed in BMMC or the more mature PCDMC in response to the TLR2 ligands MALP-2, LTA, or PGN (Mrabet-Dahbi et al., 2009). When these agonists were given i.p., no drop in body temperature was observed, suggesting that TLR2 activation does not lead to degranulation of mast cells *in vivo* (Mrabet-Dahbi et al., 2009).

It is difficult to reconcile the differences in the findings of these various studies. It may be that differences in the cell culture or isolation methods, or differences in the agonist preparations used could explain the discrepancies.

In other settings, TLR ligands have inhibited mast cell degranulation. Stimulation of LAD1 cells with dsRNA analogues resulted in decreased adhesion of the cells to fibronectin and vitronectin via TLR3, which led to a decrease in the degranulation observed when cells were allowed to adhere to these proteins (Kulka and Metcalfe, 2006). LTA and PGN acted over 24–48 h to downregulate surface levels of FcεRI expression on LAD2 cells and human lung mast cells, which resulted in a decreased degranulation after antigen exposure (Yoshioka et al., 2007). The effect was only partially mediated by TLR2 and was not observed with TLR4, 5, or 9 agonists (Yoshioka et al., 2007).

These findings suggest that TLR signaling may affect the phenotype of mast cells, for example by downregulating FcεRI expression (Yoshioka et al., 2007). Human mast cells cultured *in vitro* in the presence of LPS or PGN had altered protease composition and cytokine production profiles (Kirshenbaum et al., 2008). In another study, LPS has been shown to cause an increase in TLR4 expression in LAD2 cells, such that increased levels of TNFα were produced after a second stimulation with LPS (Kubo et al., 2007). This is in contrast to work performed on BMMC where classical endotoxin-tolerance was observed and cells were unresponsive to a second LPS challenge (Sly et al., 2004; Saturnino et al., 2010).

In conclusion, the ability of TLR2 agonists to cause mast cell degranulation is controversial and further work is required to clarify the situation. It seems that TLR stimulation affects the mast cell phenotype modulating the levels of receptors and proteases. Exposure of mast cells to TLR agonists *in vivo*, therefore, may control their ability to respond to other stimuli and the type of response they are able to mount.

## TLR SIGNALING IN THE MAST CELL

### LACK OF TRIF-DEPENDENT PATHWAY IN TLR4 SIGNALING IN THE MAST CELL

The prototypical TLR4 ligand is LPS which is bound by the secreted protein, LPS-binding protein (LBP) and transferred to the TLR4 signaling complex by cell secreted or membrane bound CD14 (Lee et al., 2012). TLR4 acts in a complex with MD-2 (Lee et al., 2012) and this has also been shown to be the case in mast cells (Ushio et al., 2004). It is now recognized that CD14 is only required for the cell to recognize rough LPS but not smooth LPS (which contains full-length *O*-chains; Jiang et al., 2005; Huber et al., 2006; Lee et al., 2012).

Conventionally, TLR4 signaling proceeds via two signaling pathways: the MyD88-dependent and the TRIF-dependent (MyD88-independent) pathways (Lu et al., 2008; Akira, 2009; Kawai and Akira, 2010). Activation of the MyD88-dependent pathway leads to the production of pro-inflammatory cytokines via activation of AP-1, IRF-5, and NF-κB. This pathway requires the adaptor protein, TIRAP, to mediate the interaction between TLR4 and MyD88. The adaptor TRAM is required for TLR4 to activate the TRIF pathway which leads to the activation of IRF-3 and, therefore, interferon-β (IFNβ) production. This pathway also causes a delayed NF-κB activation which contributes to the production of pro-inflammatory cytokines (Lu et al., 2008; Akira, 2009; Kawai and Akira, 2010).

**FIGURE 2:**
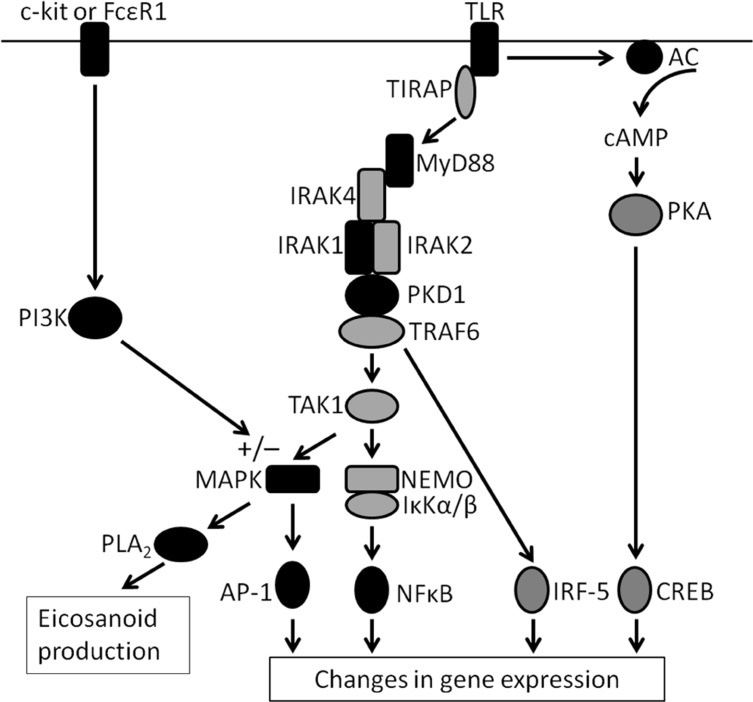
** TLR signaling in the mast cell**. A scheme illustrating the signaling pathways triggered by TLR ligation in the mast cell. Where there is evidence in the literature for the involvement of a particular protein, the protein is filled in black. Proposed molecules are in grey (adapted from Akira et al., 2006; Lu et al., 2008; Park et al., 2009). The activation of PI3K via c-kit or FcεRI stimulation is shown, with its inhibitory and activating effects on MAPK activation.

In BMMC, it appears that TLR4 signaling proceeds only via the MyD88-dependent pathway and that the TRIF-dependent pathway is not used (Keck et al., 2011). LPS stimulation of mast cells does not lead to IFN production (Dietrich et al., 2010; Keck et al., 2011) and TRIF deficiency does not affect the BMMC cytokine secretion induced by LPS (Keck et al., 2011). In addition, LPS-induced NF-κB activation is entirely dependent on MyD88 (**Figure 2**; Keck et al., 2011).

BMMC were observed to express reduced levels of TRAM, the adaptor protein that links TRIF to the TLR4 receptor complex (Keck et al., 2011). This reduction in TRAM may prevent TLR4, and therefore LPS, from activating the TRIF pathway (Keck et al., 2011). It does not appear to fully account for the defect, however, as although TRAM over-expressing BMMC produced increased IL-6 upon LPS stimulation, IFN production was still not detected (Keck et al., 2011).

TLR4 signaling via the MyD88-dependent pathway occurs from the cell membrane, whereas TRIF-mediated signaling is believed to occur in early endosomes after internalization of the TLR4 complex (Kagan et al., 2008). TLR4 is not internalized after stimulation on mast cells (Dietrich et al., 2010; Keck et al., 2011), and while in macrophages LPS is transported inside the cell, this is not the case in mast cells (Dietrich et al., 2010). It seems likely that this lack of internalization may explain why the TRIF-dependent pathway does not occur in mast cells. CD14 is required for the internalization of TLR4 (Zanoni et al., 2011; Lee et al., 2012) and although BMMC express CD14 mRNA (Ikeda and Funaba, 2003), they do not express detectable levels of CD14 on the cell surface, and CD14 must be provided in serum for the response to LPS (McCurdy et al., 2001; Varadaradjalou et al., 2003). Therefore, this lack of CD14 may explain why TLR4 is not efficiently internalized in mast cells and, therefore, why the TRIF pathway is not activated by LPS (Keck et al., 2011).

The lack of CD14 on the cell surface may not fully explain the inability of LPS to stimulate the TRIF pathway in mast cells, however, since IFNα/β production (albeit reduced) is observed upon LPS stimulation of CD14^-/-^ macrophages suggesting that CD14-independent TRIF activation is possible (Keck et al., 2011). It is not clear whether this is due to a limited degree of CD14-independent internalization of TLR4 or if the TRIF pathway is activated from the plasma membrane in this condition (Keck et al., 2011).

The inability of LPS to stimulate the TRIF-dependent pathway is not unique to mast cells. Neutrophils stimulated with LPS similarly produce no IFN (Tamassia et al., 2007). It has been suggested that the lack of TRIF signaling in response to LPS may be a protective mechanism to prevent excessive activation of mast cells by the commensal bacteria routinely encountered by the cells due to their location at sites close to the interface with the environment (Keck et al., 2011). The same may be true for other cell types and it is not known how many other cells respond in this way to TLR4 stimulation (Tamassia et al., 2007).

In addition to its key role in TLR4 signaling, CD14 is known to be involved in the responses of other TLRs to various ligands, although the molecular details are not fully understood (Lee et al., 2012). Therefore, the lack of surface CD14 may affect the response of mast cells to TLR2 and TLR5 ligands. Since CD14 mRNA has been detected in mast cells (Ikeda and Funaba, 2003) it is possible that the protein is available intracellularly and may be involved with TLR3 and TLR7–9 signaling. Further studies will be required to determine CD14 intracellular expression in mast cells and what function this protein may play in TLR signaling.

Further extending these findings that LPS treatment does not cause IFNα/β secretion, Keck et al. (2011) demonstrated that neither infection with an adenoviral vector nor B-DNA transfection, both of which stimulate macrophages to produce IFNβ, caused IFN production in mast cells. Gene array analysis of human cells found that whilst a group of interferon response genes were upregulated by LPS in monocytes, this upregulation did not occur in mast cells (Okumura et al., 2003). The lack of type I IFN production upon LPS stimulation of human mast cells was confirmed by quantitative PCR, suggesting that the IFN response to LPS is lacking in human mast cells as it is in murine mast cells (Okumura et al., 2003; Keck et al., 2011).

Mast cells are not entirely defective in IFN production, however. Infection with vesicular stomatitis virus caused IFN production (Keck et al., 2011), and polyI:C treated human and murine mast cells secrete IFNα and β (Kulka et al., 2004) although this finding was not reproduced in a later study (Keck et al., 2011). It seems that the ability of mast cells to mount a potent IFN response is tightly regulated.

### ACTIVATION OF IRAKs

The family of interleukin-1 receptor-associated kinases (IRAKs) are involved in the downstream signaling of TLRs (Akira, 2009; Kawai and Akira, 2010). Very little is known about their roles in TLR signaling in mast cells. In MC-9 cells, LPS and P3C were shown to activate IRAK1 in an *in vitro* kinase assay, suggesting that this kinase is important in signaling from TLR4 and TLR2/TLR1 (**Figure 2**; Qiao et al., 2006). Future studies are needed to address the roles played by IRAK1 other members of this family in TLR signal transduction in the mast cell.

### MAPK ACTIVATION BY TLR LIGANDS IN MAST CELLS

The MAPKs are known to play a key role in TLR signaling in immune cells (Akira, 2009; Kawai and Akira, 2010) and their role in TLR signaling in the mast cell has been addressed in several studies. The involvement of p38, Erk, and Jnk in mast cell TLR signaling has been demonstrated, although there are some discrepancies between studies. There seems to be more support for a role of p38 and Jnk in the TLR signaling pathways in the mast cell than for Erk, although some studies have identified Erk as an important player.

TLR4, TLR2/1, and TLR2/6 signaling activated p38 MAPK in BMMC in a comparable manner to stimulation through FcεRI (Zorn et al., 2009). Furthermore, an inhibitor of p38 reduced the IL-6 produced upon stimulation with LPS, Pam_3_CSK_4_, or FSL-1, suggesting that this kinase plays a role in TLR2 and 4 signaling (Zorn et al., 2009). Interestingly, inhibition of p38 phosphorylation caused a reduction in the secretion of IL-13 and IL-10 from BMMC upon LPS stimulation but did not reduce the mRNAs of these cytokines, suggesting that p38 regulates the production of these cytokines post-transcriptionally (Masuda et al., 2002).

In addition to p38 phosphorylation, Masuda et al. (2002) detected Jnk1/2 and p38 activation after LPS stimulation of MC-9 cells and BMMC which was similar to that induced by FcεRI signaling, together with a weaker Erk1/2 phosphorylation. In contrast, in a separate study, Jnk1/2 phosphorylation was not detected in BMMC after LPS stimulation (Supajatura et al., 2001). It has been suggested that this discrepancy may be explained by the sensitivity of the assays used, since Jnk1/2 activation was demonstrated in an *in vitro* kinase assay in the later study while Jnk-phosphorylation was undetectable by western blot (Masuda et al., 2002). A more recent study on BMMC revealed phosphorylation of p38, Jnk1/2, and Erk1/2 after LPS stimulation but no Erk5 phosphorylation which was induced by stimulation of FcεRI (Li et al., 2009).

Inhibition of Jnk with curcumin reduced the amount of IL-10 and IL-13 but not IL-5 produced by BMMC and MC/9 cells upon LPS stimulation, and similar results were obtained after over-expression of a dominant negative Jnk in MC/9 cells (Masuda et al., 2002). Production of the anti-microbial peptide, CRAMP upon LPS stimulation of BMMC was not dependent on the MAPK p38, Jnk1/2, or Erk (Li et al., 2009).

The IFNα production induced upon polyI:C stimulation of human PBDMC was inhibited by pharmacological inhibitors of Jnk and p38, suggesting that these pathways are also involved in TLR3 signaling in mast cells (Kulka et al., 2004). In support of these findings, Jnk and p38 phosphorylation was observed after stimulation of human PBDMC with polyI:C (Kulka et al., 2004).

Treatment of LAD2 cells with LPS, LTA, PGN, flagellin, or CpG-containing oligonucleotides resulted in phosphorylation of Erk (Yoshioka et al., 2007). In MC/9 cells, however, neither LPS nor the TLR2/TLR1 ligand, P3C was observed to cause Erk phosphorylation while both ligands induced detectable Jnk and p38 phosphorylation (Qiao et al., 2006). Specific inhibitors of all three MAPKs reduced the TNFα production induced by the ligands, suggesting that Erk does play a role in the TLR signaling in MC/9 cells, even though Erk phosphorylation was not detected (Qiao et al., 2006). In a separate study, however, pharmacological inhibition of Erk phosphorylation had no effect on the IL-5, -10, or -13 secretion induced by LPS in either MC/9 or BMMC (Masuda et al., 2002).

Pam_3_CSK_4_ induced Erk-phosphorylation in BMMC that was dependent on TLR2 and MyD88, and an inhibitor of MEK, the MAPK upstream of Erk, reduced the LTC_4_ and PGD_2_ production induced by Pam_3_CSK_4_, confirming the importance of Erk in this signaling pathway in mast cells (Kikawada et al., 2007). Sustained Erk phosphorylation was not observed in mast cells deficient for the group V secretory PLA_2_, and as a result the amount of leukotriene and prostaglandin produced upon stimulation of these cells with Pam_3_CSK_4_ was reduced (**Figure 2**; Kikawada et al., 2007). The crucial role of the PLA_2_ on signaling seems to be specific to the TLR2 pathway as the deficient mast cells responded as wild-type cells to SCF and to stimulation through FcεRI (Kikawada et al., 2007).

In conclusion, it seems that p38, Jnk, and Erk are all involved in TLR signaling in the mast cell, and that their relative predominance depends on the cells and stimuli type and concentration used, as well as the particular cytokine of interest.

### REQUIREMENT FOR ADENYLATE CYCLASE

It has been demonstrated in epithelial cells that the IL-6 production observed upon TLR4 stimulation is dependent upon the secondary messenger cAMP activating the transcription factor CREB (Song et al., 2007). Similarly in mast cells, inhibition of adenylate cyclase (AC) in CBMC reduced the IL-6 production in response to PGN and Pam_3_CSK_4_, but had no effect on the IL-1β produced (Haidl et al., 2011; **Figure 2**). This finding suggests that cAMP is important in the mast cells response to TLR2 stimulation, although interestingly, it may be redundant in the production of IL-1β (Haidl et al., 2011).

### CALCIUM SIGNALING AND PROTEIN KINASE ACTIVATION IN TLR SIGNALING IN THE MAST CELL

Calcium is not thought to be involved in TLR signaling pathways (Lu et al., 2008; Akira, 2009; Kawai and Akira, 2010), but is an important secondary messenger in the FcεRI signaling pathway in the mast cell which leads to degranulation (Gilfillan and Tkaczyk, 2006). Since some studies have demonstrated that TLR ligands cause mast cell degranulation (Supajatura et al., 2002; McCurdy et al., 2003; Varadaradjalou et al., 2003), while others have been unable to reproduce these findings (Ikeda and Funaba, 2003; Matsushima et al., 2004; Qiao et al., 2006; Mrabet-Dahbi et al., 2009; Wierzbicki and Brzezinska-Blaszczyk, 2009), it is perhaps not surprising that similar discrepancies exist in the literature describing the ability of TLR agonists to cause calcium release in mast cells.

In MC-9 cells and BMMC, LPS, PGN, MALP-2, and P3C were unable to induce calcium signaling (Qiao et al., 2006). In contrast, it has been shown in BMMC that PGN causes calcium mobilization which was dependent on TLR2, but LPS did not have this effect (Supajatura et al., 2002). As discussed, the discrepancies between these two studies are difficult to reconcile.

Protein kinase C (PKC) α and β appear to have no role in LPS signaling in the mast cell, since BMMC deficient in either kinase responded as well as wild-type cells to LPS, and an inhibitor of PKCs had only minimal effects on the response (Zorn et al., 2009).

Similarly, PKCs do not appear to play an important role in TLR2 signaling in the mast cell, since an inhibitor of PKC did not reduce the levels of CCL2/MCP-1 produced upon Pam_3_CKS_4_ stimulation of BMMC (Murphy et al., 2007). PKD1, however, was shown to be activated in BMMC upon treatment with the TLR2 agonist, Pam_3_CKS_4_ (**Figure 2**; Murphy et al., 2007). The phosphorylation of PKD1 was dependent on MyD88 and reduced levels of CCL2/MCP-1 mRNA and protein were produced by cells when a PKD inhibitor was added, suggesting that the kinase is important in the response of the cells to TLR2 ligands (Murphy et al., 2007). A more recent study in macrophages also identified a crucial role for PKD1 in Myd88-dependent TLR signal transduction (Park et al., 2009). Further work will be required to understand the roles of PKD1 and other protein kinases in TLR signaling in the mast cell.

### ROLE OF BTK IN MAST CELL TLR SIGNALING

Bruton’s tyrosine kinase (Btk) interacts with several TLR receptors and components of the TLR signaling pathway including IRAK1 and TIRAP, and the kinase is activated by LPS in THP-1 cells (Jefferies et al., 2003). The role this kinase plays in TLR signaling is controversial. In one study, mononuclear cells from patients with mutations in Btk showed an impaired TNFα response to LPS, demonstrating that this kinase is required for TLR4 signaling (Horwood et al., 2003). Whereas, another study on monocytes from patients deficient in Btk found no such defect in TLR4 signaling (Perez de Diego et al., 2006). Murine Btk^-/-^ macrophages produced reduced levels of IL-10 in response to several TLR agonists than wild-type cells, which resulted in an increase in the amount of IL-6 produced (Schmidt et al., 2006).

Btk is important in signaling through the FcεRI (Gilfillan and Tkaczyk, 2006), demonstrating that the tyrosine kinase is expressed and functional in mast cells. Btk does not appear to play a vital role in TLR signaling in the cells, however, since the response to TLR4, TLR2/TLR1, and TLR2/TLR6 ligands was either unaffected or enhanced in BTK-deficient mast cells (Zorn et al., 2009). Phosphorylation of p38 upon LPS stimulation was unaffected by Btk deficiency in BMMC (Zorn et al., 2009). These data suggest that the kinase may have an inhibitory role in TLR signaling in the mast cell, in contrast to that which has been described in monocytes and macrophages (Horwood et al., 2003; Schmidt et al., 2006; Zorn et al., 2009).

### ACTIVATION OF TRANSCRIPTION FACTORS

Roles for several transcription factors have been demonstrated in TLR activation, including AP-1-binding proteins (such as c-jun and c-fos) and NF-κB (Akira, 2009; Kawai and Akira, 2010) and some of these have been implicated in TLR signaling in the mast cell.

In BMMC, LPS and PGN caused phosphorylation of IκB-α at Ser32 (Supajatura et al., 2002) which would lead to NF-κB activation. In an earlier publication, it was demonstrated that IκB-α phosphorylation after LPS stimulation only occurred in C3H/HeN BMMC and not in BMMC derived from the C3H/HeN TLR4-mutated strain, demonstrating that this activation was induced via TLR4 (Supajatura et al., 2001), and this was supported by the lack of IκB-α phosphorylation after LPS stimulation of TLR4^-/-^ BMMC (Supajatura et al., 2002). Similarly, IκB-α phosphorylation was not observed in TLR2^-/-^ BMMC after PGN stimulation (Supajatura et al., 2002).

A more recent study detected limited IκBα degradation upon LPS stimulation and greater levels of IκBβ degradation, particularly at time points of over an hour, confirming that NFκB signaling occurs upon TLR4 signaling in mast cells (Li et al., 2009). Inhibition of this pathway reduced the levels of transcription of an anti-microbial peptide, demonstrating the importance of this pathway in the response (Li et al., 2009).

NF-κB signaling is also implicated in TLR signaling in human mast cells. IκB phosphorylation was detected in human PBDMC after polyI:C stimulation, and the IFNα induced upon polyI:C treatment of the cells was inhibited with a chemical inhibitor of NF-κB (Kulka et al., 2004). In human CBMC, PGN and Pam_3_CSK_4_ induced IL-6 and IL-1β production was inhibited by an inhibitor of IκK-2, suggesting that the NF-κB pathway is also important in TLR2 signaling in human mast cells (Haidl et al., 2011). In agreement with these findings that NF-κB is activated upon TLR2 and TLR4 stimulation of mast cells, NF-κB-binding activity was detected in nuclear extracts of MC/9 cells after stimulation with LPS and P3C (Qiao et al., 2006).

Taken together, these studies clearly define an important role for NF-κB in TLR2 and TLR4 signal transduction in human and murine mast cells (**Figure 2**). P3C and LPS treatment of MC/9 cells resulted in phosphorylation of ATF-2 and, to a lesser extent, c-Jun implying that these two transcription factors are involved in the signal transduction pathways of TLR2 and 4 in mast cells (**Figure 2**; Qiao et al., 2006). In the same study, c-fos activity was not induced by either ligand, nor was STAT 3, 5, or 6 activation detected (Qiao et al., 2006). Further work will be required to determine whether these transcription factors are activated in human and murine mast cells in addition to this cell line.

As discussed above, in stark contrast to the situation in macrophages, LPS stimulation of mast cells does not lead to IFN production (Dietrich et al., 2010; Keck et al., 2011). This is reflected in the activation of the transcription factor IRF-3 (Keck et al., 2011). In macrophages, LPS treatment causes IRF-3 phosphorylation which is not observed in BMMC, even when soluble CD14 is added to the media. When LPS is administered i.p., IRF-3 phosphorylation was observed by flow cytometry in macrophages but not mast cells, demonstrating that this difference between the two cell types also exists *in vivo* (Keck et al., 2011).

### EFFECT OF PI3K SIGNALING

Phosphoinositide 3-kinase (PI3K) is composed of a p110 catalytic subunit and a p85 regulatory subunit and its action produces lipid mediators which act as secondary messengers and activate downstream kinases. The PI3K pathway is a regulator of TLR signaling which can have either positive or negative effects on signaling depending on cell type and stimulus, as reviewed by Hazeki et al. (2007).

Since PI3K activation leads to phosphorylation of the kinase AKT, AKT phosphorylation can be used as a readout for PI3K activation. AKT phosphorylation was not detected after LPS or P3C activation of MC/9 cells, suggesting that these TLR4 and TLR2 ligands do not activate the PI3K pathway in these cells (Qiao et al., 2006).

Inhibition of the PI3K pathway with two pharmacological inhibitors reduced the amount of TNFα, IL-6, and IL-1β produced by BMMC upon LPS stimulation (Sly et al., 2004). In a more recent study, however, whilst Wortmannin reduced the amount of TNFα and IL-6 produced while the IL-1β production was increased, suggesting that the pathway differentially regulates cytokine production in mast cells (Hochdorfer et al., 2011). These disparate findings regarding the role of PI3K signaling in IL-1β production are difficult to reconcile, particularly since both studies used BMMC and similar concentrations of Wortmannin (Sly et al., 2004; Hochdorfer et al., 2011). Co-treatment of cells with LPS and known PI3K stimulating factors such as IGF-1 caused an increase in the amount of TNFα produced, but inhibited the production of IL-1β in murine BMMC and PCDMC (Hochdorfer et al., 2011; **Figure 2**).

The differential effects of PI3K activation on TNFα and IL-1β is intriguing, and a similar result was obtained in human monocytes, in that the inhibition of PI3K differentially affected the production of two cytokines (Martin et al., 2003). When monocytes were stimulated with LPS in the presence of PI3K inhibitors, the amount of IL-12 produced was increased whilst the amount of IL-10 produced was inhibited (Martin et al., 2003). The mechanism behind the disparity appears to be that inhibition of PI3K led to suppression of Erk1/2 activation, and Erk has been previously demonstrated to cause the production of IL-10 and suppress IL-12 production in RAW264.7 cells (Yi et al., 2002). Perhaps a similar mechanism is at work in mast cells, and may explain the opposing effect that PI3K activation has on TNFα and IL-1β production.

Activation of the SCF receptor, c-kit, potently induces PI3K signaling in mast cells, and there are several mutations of the c-kit receptor which are associated with human disease that result in constitutive c-kit activation (Robyn and Metcalfe, 2006). This raised the interesting possibility that mast cells in patients with particular c-kit mutations may respond differently to stimulation with LPS. Indeed, the L138.8A mast cell line which contains such a c-kit activating mutation did not produce IL-1β upon LPS stimulation unless PI3K signaling was chemically inhibited (Hochdorfer et al., 2011). It is interesting to speculate that mast cell responses to TLR agonists *in vivo* may be modulated by other stimuli that the cell encounters that activate the PI3K signaling pathway.

## ACTIVATION OF INHIBITORY PATHWAYS

Several pathways that inhibit TLR signaling have been identified which presumably act to prevent over-reaction of cells to TLR ligands which could result in immune-mediated pathology (Kawai and Akira, 2010). The presence of some of these pathways has been investigated in mast cells, and several have been shown to be functional in the cells.

In macrophages, TIRAP becomes phosphorylated and degraded by SOCS1 after TLR2 and TLR4 activation, which consequently prevents further signaling via the MyD88-dependent pathway (Mansell et al., 2006). This inhibitory pathway does not occur in mast cells, however, and the levels of TIRAP remain unchanged after stimulation (Zorn et al., 2009). In mast cells, LPS activation leads to a reduction in the levels of mRNA of SOCS1 and CISH (a SOCS family member), whereas in macrophages it results in an increase in the levels of SOCS1, SOCS3, and CISH, which are thought to be responsible for the degradation of TIRAP (Mansell et al., 2006; Zorn et al., 2009).

The SH2-containing inositol phosphatase (SHIP) inhibits the NF-κB pathway during FcεRI stimulation of mast cells (Kalesnikoff et al., 2002) and has been shown to be upregulated in both mast cells and macrophages after LPS stimulation (Sly et al., 2004). The ability of SHIP to negatively regulate TLR4 signaling is illustrated by the demonstration that injection of a sub-lethal concentration of LPS was lethal in SHIP deficient animals (Sly et al., 2004). SHIP inhibits signaling through the PI3K pathway (Huber et al., 1998) and since inhibition of PI3K inhibits the LPS-induced cytokine production in mast cells (Sly et al., 2004; Hochdorfer et al., 2011), it seems logical that SHIP would inhibit TLR4 signaling in these cells. Indeed, SHIP-mediated negative feedback has been shown to be important in the phenomenon of endotoxin-tolerance in both mast cells and macrophages, since endotoxin-tolerance could not be induced in SHIP^-/-^ mast cells or macrophages (Sly et al., 2004). The SHIP expression in LPS stimulated mast cells and macrophages is caused by autocrine TGFβ, implying that this regulatory cytokine is important in inhibiting the response to LPS in both cell types (Sly et al., 2004).

As previously discussed, inhibition of the PI3K pathway in mast cells does not inhibit all cytokine production stimulated by LPS. Rather, the production of IL-1β was enhanced when the pathway was inhibited (Hochdorfer et al., 2011). In support of this, whilst LPS stimulation of SHIP^-/-^ mast cells resulted in greater TNFα production, the amount of IL-1β secreted was reduced (Hochdorfer et al., 2011).

DAP12 is a transmembrane protein which has been shown to inhibit the response of macrophages to TLR agonists (Hamerman et al., 2005). DAP12^-/-^ BMMC, however responded to TLR4, TLR2/TLR1, and TLR2/TLR6 agonists in a comparable manner to wild-type cells, suggesting that the signaling pathways in mast cells are independent of DAP12 (Smrz et al., 2010).

TANK is a negative regulator of TLR signaling (Kawagoe et al., 2009) and has been identified in gene array analysis as being up-regulated in LPS stimulated mast cells (Okumura et al., 2003), raising the possibility that it may be involved in a negative feedback loop. Further work will be required to determine whether this protein indeed inhibits TLR signaling, and to establish what other pathways are important in the regulation of TLR-mediated mast cell activation.

Other as yet unidentified mechanisms may be in place to limit the response of the mast cell to LPS, or perhaps, since this does not result in IFN production (Dietrich et al., 2010; Keck et al., 2011), regulation of the mast cell LPS response is less crucial than that of macrophages. Indeed, it has been suggested that the lack of TRIF signaling in response to LPS may be a protective mechanism to prevent excessive activation of mast cells by the commensal bacteria routinely encountered by the cells due to their location at sites close to the interface with the environment (Keck et al., 2011).

## RECEPTOR CROSS-TALK BETWEEN TLRs AND OTHER RECEPTORS ON MAST CELLS

Dectin-1 is a PPR which is known to interact with several TLRs, and behaves as a co-receptor for TLR2 (Gantner et al., 2003; Reid et al., 2009). Dectin-1 is believed to be primarily expressed on myeloid cells, and has been shown to be expressed on human mast cells (Olynych et al., 2006). Inhibition of dectin-1 reduced the LTC_4_ produced upon CBMC stimulation with zymosan but not PGN, and did not inhibit the production of GM-CSF or IL-1β (Olynych et al., 2006). These data suggest that the PPR is involved in mast cell recognition of zymosan, presumably in conjunction with TLR2, but not of PGN, and that the receptor is required for cell signaling to induce the production of lipid mediators but not cytokines (Olynych et al., 2006). In support of this, pharmacological inhibition of the tyrosine kinase Syk, which is activated by dectin-1, inhibited the production of LTC_4_ induced by zymosan and to a lesser extent by PGN. Syk is therefore important in the downstream signaling from TLR2 in mast cells (Olynych et al., 2006).

Stimulation of mast cells via TLRs results in cytokine and chemokine production in a similar way to that observed for other cells (Akira et al., 2006; Kawai and Akira, 2010). Mast cells are unique in that they express the FcεRI in addition to TLRs, and so there is the potential for cross-talk between these two cell stimulatory pathways. Several studies have addressed the impact of TLR signaling on stimulation of mast cells via the FcεRI and vice versa.

Mast cells sensitized with IgE respond more robustly to LPS stimulation (Medina-Tamayo et al., 2011). This enhanced sensitivity is not due to an increase in the expression of TLR4, CD14, or MD-2, rather the cells appear to be “pre-activated” by binding IgE and show higher basal levels of NF-κB activation (Medina-Tamayo et al., 2011). This finding adds to earlier demonstrations that IgE binding to the FcεRI activates mast cells to some extent (Kawakami and Kitaura, 2005). In addition, the anti-apoptotic effect of monomeric IgE on mast cells is synergistically enhanced by the addition of LPS, signaling via TLR4, although LPS alone had no effect on apoptosis (Jayawardana et al., 2008).

In addition to these effects, the FcεRI signaling pathway shares many features with TLR signaling, for example both pathways utilize MyD88 (Gilfillan and Tkaczyk, 2006; Akira, 2009; Kawai and Akira, 2010), therefore there is potential for cross-talk between the two pathways (Avila and Gonzalez-Espinosa, 2011).

The cytokine production of BMMC and MC/9 cells upon stimulation via the FcεRI receptor is synergistically enhanced in the presence of the TLR4 agonist, LPS, and the TLR2/TLR1 agonist P3C, and to a lesser extent by that of MALP-2 and PGN (both TLR2/TLR6 agonists; Qiao et al., 2006). In contrast, the degranulation response is unaffected (Qiao et al., 2006).

Stimulation of BMMC with the TLR2 ligands MALP-2 and Pam_3_CSK_4_ synergizes with stimulation through FcεRI to enhance IL-6 production (Fehrenbach et al., 2007). MALP-2 had no effect on FcεRI-induced degranulation whereas Pam_3_CSK_4_ inhibited antigen-induced degranulation, although this was found to be due to a direct interaction between the model antigen and the lipid itself, rather than any cross-talk between signaling pathways (Fehrenbach et al., 2007).

Signaling through FcεRI induces PI3K activation in mast cells (Yano et al., 1993; Gilfillan and Tkaczyk, 2006) and, as previously discussed, inhibition of the PI3K pathway during LPS stimulation results in an inhibition of IL-6 and TNFα (although the effect on IL-1β production is contentious), suggesting that this pathway acts to increase responses to LPS (Sly et al., 2004; Hochdorfer et al., 2011; **Figure 2**). In agreement with this, stimulation of BMMC via FcεRI enhances the IL-6 and TNF induced by LPS, whilst inhibiting the IL-1β production (Hochdorfer et al., 2011).

In MC/9 cells, synergy has also been observed between TLR4 and FcεRI induced Jnk and p38, but not Erk phosphorylation (Masuda et al., 2002; Qiao et al., 2006). The use of selective MAPK inhibitors, however, did suggest a role for Erk signaling in the synergy between the two signaling pathways (Qiao et al., 2006). In contrast, Smrz et al. (2010) found no evidence of synergy between FcεRI and TLR signaling in the activation of p38, Erk, or Jnk. The activation of the transcription factors, ATF-2, c-Jun, and c-Fos upon stimulation via FcεRI was increased in the presence of either TLR2 or TLR4 stimulation (Qiao et al., 2006).

Synergy was not observed for the calcium response induced by antigen, indeed, P3C was shown to inhibit the calcium release induced by mast cell activation via the FcεRI pathway by an unknown mechanism (Qiao et al., 2006). Similarly, the IRAK1 activation caused by the TLR ligands was slightly inhibited by antigen stimulation (Qiao et al., 2006).

Synergy between TLR and FcεRI signaling pathways therefore has been demonstrated (Qiao et al., 2006; **Figure 2**), and results in enhanced cytokine secretion but not degranulation (Qiao et al., 2006; Fehrenbach et al., 2007; Hochdorfer et al., 2011). Much of this work has been performed in murine mast cells and it would be interesting to investigate the phenomenon in the human context. It has been proposed that the increased response to stimulation via FcεRI in the presence of TLR2 and 4 ligands may contribute to the worsening of allergic symptoms which can occur in the presence of pathogens (Qiao et al., 2006).

## CONCLUSION

Recent research identifies important roles for mast cells in the immune defense against bacteria and pathogens (Marshall, 2004; Abraham and St John, 2010) and given their locations at sites of microbial entry into the host (Metcalfe et al., 1997) the ability of the cells to recognize invading pathogens must be crucial. A variety of PRRs are responsible for initial recognition of pathogens (Akira et al., 2006) and of these, the TLRs are the best studied in mast cells.

Mast cells have been shown to express the majority of TLRs (**Table 2**) and respond to their agonists by secreting cytokines, chemokines and lipid mediators which would have a profound effect on other cells of the immune system. In addition, TLR ligation can act to enhance the response of mast cells to antigen, sensitizing the cells to stimulation through FcεRI (Qiao et al., 2006; Fehrenbach et al., 2007; Hochdorfer et al., 2011). To date, the majority of the work investigating the function of TLRs has been performed *in vitro* with only a few studies *in vivo* (Supajatura et al., 2002; Mrabet-Dahbi et al., 2009). Further studies are therefore required to fully elucidate the role of TLR signaling in mast cells.

The signal transduction pathways triggered by TLR stimulation of mast cells are beginning to be elucidated and have some unique features. Strikingly, the MyD88-independent pathway which leads to IFN production is not induced by TLR4 activation, which may be due to a lack of cell surface CD14 (Keck et al., 2011). Indeed, the ability of mast cells to secrete IFN in response to other TLR stimulation is somewhat controversial (Kulka et al., 2004; Keck et al., 2011). In addition, some of the inhibitory pathways which have been identified in other immune cells are not observed to occur in mast cells (Hamerman et al., 2005; Zorn et al., 2009).

As described, several discrepancies are noted in the literature reporting the signaling pathways utilized by TLRs in mast cells and the response to TLR agonists. Notably, the ability of TLR2 ligation to induce degranulation is contentious, as is the ability of mast cells to respond to TLR3 ligation and the relative importance of the different MAPK proteins in TLR signal transduction. These differences may be explained by different mast cell culture conditions resulting in heterogeneous cell populations possibly with different expression of TLRs and signaling proteins. Further work is needed to consolidate the data. Given the importance of mast cells in the immune defense to bacteria and viruses (Marshall, 2004; Abraham and St John, 2010), it is important that the signal transduction pathways utilized by TLRs and the consequences of TLR signaling in these cells are understood.

## Conflict of Interest Statement

The authors declare that the research was conducted in the absence of any commercial or financial relationships that could be construed as a potential conflict of interest.

## Abbreviations

AC: adenylate cyclase
BMMC: bone marrow-derived mast cell
Btk: Bruton’s tyrosine kinase
CBMC: cord blood-derived mast cell
cysLT: cysteinyl leukotriene
FSDMC: fetal skin-derived mast cells
IFN: interferon
IGF-1: insulin-like growth factor-1
IHC: immunohistochemistry
LBP: LPS-binding protein
MD-2: myeloid differentiation-2
n.d.: not determined
P3C: tripalmitoyl Cys-Ser-(Lys)4
PAMPs: pathogen-associated molecular patterns
PBDMC: peripheral blood-derived mast cell
PCDMC: peritoneal cell-derived mast cell
PI3K: phosphoinositide 3-kinase
PKC: protein kinase C
